# Sex and early-life conditions shape telomere dynamics in an ectotherm

**DOI:** 10.1242/jeb.246512

**Published:** 2024-02-09

**Authors:** Alexander Hansson, Erik Wapstra, Geoffrey M. While, Mats Olsson

**Affiliations:** ^1^ Department of Biological and Environmental Sciences, University of Gothenburg, Box 463, 405 30 Gothenburg, Sweden; ^2^ School of Natural Sciences, University of Tasmania, Private Bag 55, Hobart, TAS 7001, Australia

**Keywords:** Telomere dynamics, Development, Incubation temperature, Life history, Sexual dimorphism, Reptile

## Abstract

Telomeres, the repetitive DNA regions that protect the ends of chromosomes, and their shortening have been linked to key life history trade-offs among growth, reproduction and lifespan. In contrast to most endotherms, many ectotherms can compensate for telomere shortening throughout life by upregulation of telomerase in somatic tissues. However, during development, marked by rapid growth and an increased sensitivity to extrinsic factors, the upregulation of telomerase may be overwhelmed, resulting in long-term impacts on telomere dynamics. In ectotherms, one extrinsic factor that may play a particularly important role in development is temperature. Here, we investigated the influence of developmental temperature and sex on early-life telomere dynamics in an oviparous ectotherm, *Lacerta agilis*. While there was no effect of developmental temperature on telomere length at hatching, there were subsequent effects on telomere maintenance capacity, with individuals incubated at warm temperatures exhibiting less telomere maintenance compared with cool-incubated individuals. Telomere dynamics were also sexually dimorphic, with females having longer telomeres and greater telomere maintenance compared with males. We suggest that selection drives this sexual dimorphism in telomere maintenance, in which females maximise their lifetime reproductive success by investing in traits promoting longevity such as maintenance, while males invest in short-term reproductive gains through a polygynous mating behaviour. These early-life effects, therefore, have the potential to mediate life-long changes to life histories.

## Teaser

**Highlighted Article:** Telomere dynamics are strongly sexually dimorphic and influenced by developmental temperature in neonate sand lizards (*Lacerta agilis*).

## INTRODUCTION

Telomere dynamics, the length and change of telomeres across time, represent a balance between telomere shortening and elongation processes crucial to organismal health. Extreme rates of either can be detrimental to fitness. For example, excessive shortening is linked to a loss of cellular homeostasis and diminished survival at both the cellular and organismal (and even population) level (Dupoué et al., 2017, 2022; Young, 2018), whereas high rates of elongation, via increased telomerase activity, raise the risk of immortalising cancerous cells (Aviv et al., 2017). Organisms have found different strategies to balance these processes. In endotherms, telomerase is typically repressed in somatic tissues, whereas it often remains upregulated in somatic tissues throughout life in ectotherms (Gomes et al., 2010), although the meagre available data suggest its activity varies greatly among species (Olsson et al., 2018a). As a result, telomere dynamics are diverse across ectotherms in which telomeres may shorten with age in some species while remaining unchanged, or even lengthening, in others (reviewed in Bronikowski, 2008; Simide et al., 2016; Olsson et al., 2018a; Burraco et al., 2020; see also Fitzpatrick et al., 2021).

In ectotherms capable of circumventing telomere shortening in adulthood, the majority of telomere attrition may be confined to ontogeny, e.g. embryonic development during which extreme rates of growth and cellular turnover may overwhelm telomere-restoring processes (Hatakeyama et al., 2008, 2016; Debes et al., 2016; McLennan et al., 2016). Moreover, organisms are often most sensitive to extrinsic factors during ontogeny – first and foremost temperature in ectotherms – often manifesting in long-lasting reaction norms including telomere dynamics (reviewed in Lindström, 1999; Metcalfe and Monaghan, 2001; Friesen et al., 2022). Therefore, assuming that telomere maintenance is a costly activity, as an investment in future reproductive success, it will compete for resources early in life with traits promoting current growth and early reproduction. Telomeres, along with the early-life conditions that influence them, may therefore serve as critical ecological markers of life history trajectories with significant implications for the evolution of species and populations. Critically, such effects are likely to be sex specific. One of the reasons for this is that, in ectotherms, females typically have increased fecundity with age and size, meaning that the relative contribution to overall fitness of reproductive events increases with age (Hoekstra et al., 2020). In such cases, selection should favour a longer lifespan by investing more resources in the soma, including telomeres (Vaupel et al., 2004). In contrast, selective pressures may drive polygynous males towards faster growth, early maturation and sexual competition including secondary sexual traits, at a cost to somatic maintenance and lifespan (Clutton-Brock and Isvaran, 2007; Dammhahn et al., 2018). Sex-specific telomere dynamics have been shown in adulthood in the oviparous sand lizard (*Lacerta agilis*), with females having longer telomeres and receiving greater fitness benefits from telomere maintenance compared with males (Olsson et al., 2011a). This is predicted by life history theory as female fecundity increases with age/size in this species (Olsson and Shine, 1996), and selection should therefore favour greater investment in somatic maintenance and lifespan in this sex. This is further supported by females being the longer-lived sex (Olsson, 1988; Strijbosch and Creemers, 1988). In this study, we used *L. agilis* to investigate the influence of developmental temperature and neonate growth on telomere dynamics early in life, and we also tested whether telomere dynamics are sex specific. We did this to determine how early-life environments and sex effects may influence telomeres in an ectothermic species in which telomeres are linked to fitness traits in a sex-specific manner.

## MATERIALS AND METHODS

### Study species and experimental design

The sand lizard (*Lacerta agilis* Linnaeus 1758) is a small (maximum 20 g) ground-dwelling oviparous lizard with one of the largest distribution ranges of any reptile (Bischoff, 1984). We captured gravid females over 3 years (2018–2020) from an isolated small island population on the Swedish west coast (57°29′N, 11°56′E), near the northern limit of the species' distribution. Females were individually housed in cages (500×400×350 mm) on sand substrate with a 40 W spotlight aimed at a flat basking rock, and an ambient temperature set to fluctuate daily between 15 and 20°C. Water was available *ad libitum* and individuals were fed daily with mealworms dusted with calcium and multivitamin supplements. The gravid females were closely monitored for signs of oviposition.

Once a clutch was laid, a whole-blood sample was taken from the mother for later telomere analysis by puncturing the vena angularis in the corner of the mouth with a 50 µl capillary tube. Eggs were weighed (±0.01 g) and individually placed in plastic cups half-buried in moist vermiculate (1:8 water to vermiculite by volume). We divided sibling eggs among three constant temperature incubators (23, 25 and 27°C) in accordance with a split-brood design which minimises the risk of confounding parental and treatment effects (Via, 1993). Developmental temperatures were based around 25°C, which is the optimal developmental temperature of *L. agilis* that minimises developmental abnormalities and asymmetries (Zakharov, 1989). Eggs were rotated weekly within each incubator to minimise thermal gradient effects. Hatchlings were removed within hours of hatching and measured for snout–vent length (SVL) (±1 mm) and a tail tip tissue sample was taken for later telomere analysis. Hatchlings were also sexed at this time by hemipene eversion (Harlow, 1996; Olsson and Shine, 2003), a method with 100% repeatability in this species (Olsson et al., 2004, 2005, 2011b). This incubation experiment was repeated for three consecutive years (2018–2020; see Table S1 for the complete dataset).

To examine the influence of growth and the prolonged effects of thermal treatment on neonate telomere dynamics, we also resampled tissues from neonates after a period of growth. This was only undertaken in 2019–2020. Neonates were housed under the same conditions as their mothers, but with additional basking rocks. Initially, a maximum of 10 neonates were housed per cage, which was reduced to five after an initial growth period of 2 weeks. They were fed small crickets (2–6 mm) and mealworms daily with the same supplements as adults. Leftover feed was maintained in the cage, allowing neonates to feed *ad libitum*. Water was also available in the same manner and cages were misted daily to provide additional moisture. At the end of the experiment, we remeasured and resampled tissues from neonates that had grown for between 3 and 7 weeks (35.9±0.8 days). All tissue samples were immediately placed in ethanol and stored in −30°C until DNA extraction. DNA extraction and telomere analyses of all samples were performed simultaneously in 2022. Tissue samples (*n*=150) from neonates were then randomly selected for telomere analyses and use in this study, but following the criteria of (1) a maximum of one offspring per mother within each thermal treatment and (2) a balanced selection of male and female offspring within thermal treatments.

### Telomere analyses

DNA was extracted from whole blood in PBS (replacing ethanol before extraction) and tail tissue by first adding a lysis buffer and proteinase K mixture. Samples were then homogenised with a steel bead in a bead mill (TissueLyser, Qiagen) oscillating at 25 Hz for 30 s for blood and 4 min for tail tissues, after which RNase A was added and samples were incubated for 30 min at 60°C and vortexed every 10 min. Each extraction batch included an extraction negative control (ENTC). From the lysed homogenates, DNA was isolated using the Mag-Bind Blood & Tissue DNA HDQ Kit (cat no. M6399-01, Omega Bio-tek) using a KingFisher Flex automated extraction system (Thermo Fisher Scientific). To evaluate DNA concentration and purity, we used a Lunatic spectrophotometer (Unchained Labs). DNA integrity was measured with capillary gel electrophoresis (Fragment Analyzer, Agilent Technologies Inc.) using the Genomic DNA 50 kb Kit (cat no. DNF-467-0500, Agilent Technologies Inc.). DNA samples were then stored at −80°C.

Telomere length was measured using real-time quantitative PCR (qPCR) run in 20 µl reaction volumes on a Quantstudio 7 Pro (Applied Biosystems) qPCR system. Our protocol is based on Cawthon's (2002) method optimised for *L. agilis* and the tissues used in this study (see Rollings et al., 2019; Axelsson et al., 2020; Fitzpatrick et al., 2021; Olsson et al., 2022). We used the previously published telomere primers Tel1b (5′ CGGTTTGTTTGGGTTTGGGTTTGGGTTTGGGTTTGGGTT 3′) and Tel2b (5′ GGCTTGCCTTACCCTTACCCTTACCCTTACCCTTACCCT 3′) (Criscuolo et al., 2009) and the control single-copy gene (glyceraldehyde 3-phosphate dehydrogenase, GAPDH) was amplified using the primers GAPDH-F (5′ AACCAGCCAAGTATGATGACAT 3′) and GAPDH-R (5′ CCATCGGCAGCTGCCTTCA 3′). Each reaction included 2 ng DNA, 10 µl TATAA SYBR GrandMaster Mix Low Rox (TATAA Biocenter) and 10 µmol l^−1^ primers (telomere: 0.2 µl and GAPDH: 0.8 µl), with all pipetting performed by an OT-2 pipetting robot (Opentrons). Amplifications were carried out using an initial activation step at 95°C for 3 min and a total of 40 cycles of 95°C for 15 s, 58°C (60°C for GAPDH assays) for 60 s. To evaluate the optimum annealing temperature for the telomere and the GAPDH assay, temperature gradients ranging from 55 to 65°C were run on CFX96 and CFX384 (Bio-Rad) PCR detection systems. A melt curve was created after each run over the temperature range of 60 to 95°C to ensure no non-specific product amplification. All neonate tissue samples, including tail tip samples at hatching and post-growth, were randomised among plates and run together with a no-template control (NTC) and a ‘golden standard’ (control sample) in triplicate, all with an accepted standard deviation of ≤0.5. Standard curves were created using the pooled DNA from nine randomly selected neonate individuals for both telomeres and GAPDH using a 4-fold dilution series to ensure consistent amplification across DNA concentrations (25–0.00625 ng µl^−1^). The average primer efficiencies were 87% for GAPDH reactions and 89% for telomere reactions with average *R*^2^-values both above 0.99. Telomere and GAPDH reactions were run on separate plates, including a total of nine plates for telomere reactions and three plates for GAPDH reactions. The higher number of telomere plates is explained by the commercial lab running an additional proprietary single copy gene on these plates (not further reported on here). The average standard deviation in Cq-values between triplicates was 0.14 for telomere reactions and 0.06 for GAPDH reactions. We corrected for plate effects by including an Inter-Plate Calibrator (IPC, TATAA Biocenter) run in four replicates (intra-plate standard deviation <0.04), and using the formula:
(1)

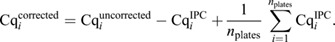
Relative telomere length (rTL) was then calculated for each sample as the ratio (*T*/*S*) between the telomere repeat copy number (*T*) and the reference single-copy gene number (*S*), following the formula:
(2)

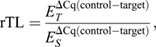
where *E*=10^−1/slope^ and slope is determined from the standard curve of each assay, and ΔCq is the difference in Cq values between the control DNA sample and the target DNA sample (Pfaffl, 2001).

### Data analysis

All statistical models were fitted in R v.4.2.2 (http://www.R-project.org/). Linear mixed models (LMM) were fitted using the *lmer* function from the *lme4* package (Bates et al., 2014), in which denominator degrees of freedom, statistics and *P*-values were derived based on the Kenward–Roger approximation using the *lmerTest* package (Kuznetsova et al., 2017). Maternal identity and plate number (when applicable) were included as random effects in all mixed models to account for pseudo-replication of siblings and plate effects. Year was not significant in any model and resulted in poor model fit, and was, therefore, not included as a predictor variable in any final model. No model revealed any significant sex-by-treatment effects and this interaction term was therefore excluded from all models. To determine general effects of incubation treatment and offspring sex, we fitted three LMMs with those factors as predictors. Incubation duration (number of days from oviposition to hatching), egg mass and SVL at hatching were included as response variables.

To identify predictors of telomere length at hatching, we fitted a LMM with initial telomere length as response, and treatment temperature, sex and maternal telomere length as fixed factors. Initial telomere length was log-transformed to meet residual assumptions. We also examined neonate telomere dynamics during early growth by fitting a LMM with daily change in telomere length [calculated as: (post-growth telomere length−initial telomere length)/number of growth days] as response. We included treatment temperature, sex, initial telomere length and maternal telomere length as predictors. Because the analysis examined the effect of initial telomere length on a subsequent measurement of telomere length, we corrected for the statistical artifact of regression to the mean prior to calculating the change in telomere length following the method developed by Verhulst et al. (2013) with a minor modification by Hoelzl et al. (2016), calculating the corrected value (*D*) as:
(3)

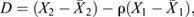
where
(4)

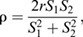
in which *X*_1_ is baseline telomere length, *X*_2_ is subsequent telomere length, *S*_1_ and *S*_2_ are the standard deviations of *X*_1_ and *X*_2_, and *r* is the correlation between *X*_1_ and *X*_2_. We also computed Tukey pairwise comparisons of among-treatment differences in change in telomere length using the *emmeans* function (https://CRAN.R-project.org/package=emmeans). Because telomere and GAPDH assays, and initial and post-growth samples were run on different plates, we compared model fit with the different plate variables included as random effects. For the initial telomere length model, telomere plates showed the best fit (AIC_no plate ID_=142.22, AIC_telomere_=70.04, AIC_GAPDH_=102.48) and we therefore used this as a random effect in the final model. The daily change of telomere length model had four possible plate effects to account for, including telomere/GAPDH assays at hatching and telomere/GAPDH post-growth. Using GAPDH (post-growth) plates as random effect yielded the best fit (AIC_no plate ID_=−284.64, AIC_telomere(initial)_=−282.64, AIC_GAPDH(initial)_=−282.64, AIC_telomere(post-growth)_=296.18, AIC_GAPDH(post-growth)_=−302.57) and was used in the final model. Finally, we examined whether growth, as a proxy for the number of cell divisions and oxidative stress, negatively impacted telomere length. We did this by fitting a LMM with absolute change in telomere length (calculated as: post-growth telomere length−initial telomere length) as response and absolute growth (calculated as: post-growth SVL−SVL at hatching) as predictor, including treatment and sex as covariates. An interaction term between absolute growth and treatment was also included, but resulted in a poorer model fit and therefore was excluded.

## RESULTS

Incubation duration was inversely related to treatment temperature, while there was no difference in incubation duration between female and male offspring (LMM: treatment, *F*_2,78.85_=965.34, *P*<0.0001; sex, *F*_1,91.86_=0.278, *P*=0.599). Eggs incubated at 23°C hatched after an average of 50.2±0.4 days (44–55 days), 25°C-incubated eggs after 38.0±0.5 days (33–43 days), and 27°C-incubated eggs after 34.0±0.31 days (32–38 days). The mass of eggs did not differ among thermal treatments or between female and male offspring (LMM: treatment, *F*_2,75.56_=0.255, *P*=0.776; sex, *F*_1,80.80_=0.578, *P*=0.450). There was no influence of treatment temperature on hatchling SVL, but female offspring had significantly longer SVL at hatching compared with males (LMM: treatment, *F*_2,78.91_=0.728, *P*=0.486; sex, *F*_1,92.10_=29.19, *P*<0.0001). The average SVL was 29.56±0.15 mm in females and 28.61±0.16 mm in males.

**Fig. 1.**
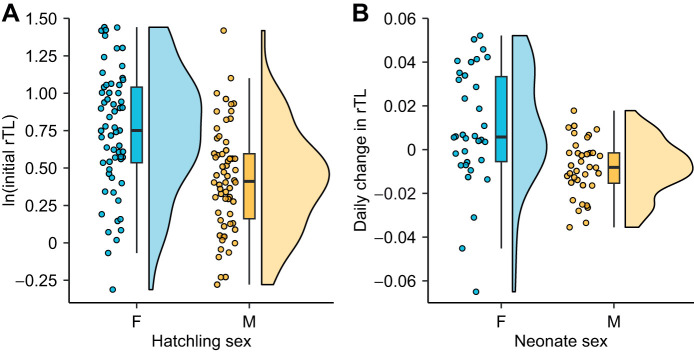
**Sex-specific telomere dynamics in hatchling and neonatal *Lacerta agilis*.** (A) The variation in initial relative telomere length (rTL) of female and male hatchlings (*n*=61 females, *n*=62 males) and (B) the variation in daily change in rTL, corrected for regression to the mean, of female and male neonates (*n*=34 females, *n*=36 males). Hatchling telomere lengths were log-transformed.

There was a significant difference in telomere length at hatching between the sexes, with females having, on average, 34% longer telomeres compared with males (Table 1 and Fig. 1A). There was no effect of either treatment temperature or maternal telomere length on telomere length at hatching (Table 1). The change in neonate telomeres was on average positive in females and negative in males, and females also showed about 2 times greater variation in telomere change compared with males (Fig. 1B). Specifically, telomere length changed at a rate that after 60 days would correspond to a mean increase of 7.1% in females and a mean decrease of 11.5% in males (Table 2, Fig. 1B). Absolute change in neonate telomere length was not associated with an absolute increase in SVL (LMM; *t*_63.52_=0.465, *P*=0.644).

**Table 1.**
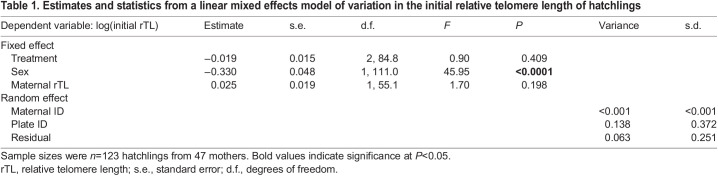
Estimates and statistics from a linear mixed effects model of variation in the initial relative telomere length of hatchlings

**Table 2.**
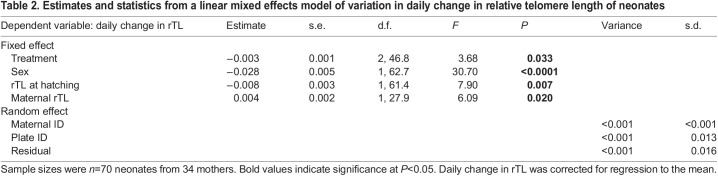
Estimates and statistics from a linear mixed effects model of variation in daily change in relative telomere length of neonates

**Fig. 2.**
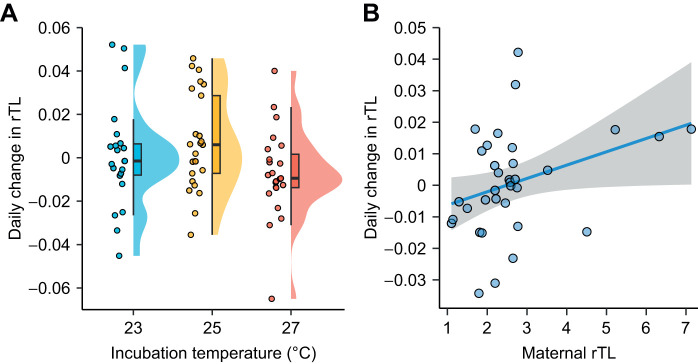
**Effects of treatment temperature and maternal telomere length on daily change in rTL of neonates.** (A) Change in rTL for the three thermal treatments (sample sizes were *n*=22, 25 and 23 for 23°C, 25°C and 27°C, respectively). (B) Change in neonate rTL was positively correlated with maternal rTL, in which plot points represent sibling averages in cases when individuals share a mother. A linear regression line (blue) through the data is shown with 95% confidence intervals (grey shading). Telomere length was corrected for regression to the mean.

Change in telomere length was overall inversely related to developmental temperature irrespective of sex (Table 2 and Fig. 2A). The average change in telomere length was negative in neonates from the warmest treatment, while it was positive in neonates from the two cooler treatments, suggesting a curvilinear relationship (Fig. 2A). Pairwise Tukey comparisons revealed that the difference in telomere change in neonates between the warmest treatment and each of the two cooler treatments was marginally significant (25–27°C: β=0.0115±0.0048, *t*-ratio_46.0_=2.406, *P*=0.0519; 23–27°C: β=0.0114±0.0050, *t*-ratio_49.0_=2.283, *P*=0.0677; 23–25°C: β=−0.0001±0.0049, *t*-ratio_45.6_=−0.024, *P*=0.9997).

The change in telomere length was positively related to maternal telomere length; mothers with longer telomeres produced offspring that better maintained their telomeres compared with those from mothers with shorter telomeres (Fig. 2B). Furthermore, when correcting for regression to the mean effects, the model still showed that change in telomere length was significantly related to initial telomere length, with shorter initial telomeres being better maintained/elongated compared with longer telomeres at hatching (Table 2).

## DISCUSSION

We examined the influence of developmental temperature, sex and maternal telomere length on early-life telomere dynamics in the sand lizard, *Lacerta agilis*. There was no evidence that developmental temperature influenced telomere length at hatching. This was surprising given that temperature directly influences the rate of biological processes in ectotherms, which is predicted to influence telomere dynamics via increased oxidative stress and an elevated metabolism that is associated with growth (Monaghan and Ozanne, 2018; Friesen et al., 2022). However, as developmental temperature strongly predicted incubation duration in *L. agilis* embryos, with no difference in hatchling body size among thermal treatments, the total number of cells, and thus cell divisions during embryogenesis, is likely to be comparable among thermal treatments (see Booth et al., 2000, for temperature and total oxygen consumption). This would remove a key mechanism by which developmental temperature could cause differential telomere erosion, explaining the lack of differences in initial telomere length between thermal treatments. Alternatively, any thermally induced telomere shortening occurring in the embryo through cell proliferation and/or oxidative damage is compensated for by the activity of telomerase or other telomere lengthening processes. Comparative studies of developmental effects on embryonic telomere dynamics in ectotherms are still too few to make generalisations (but see McLennan et al., 2018; Bae et al., 2021). Recent studies in birds – which share some developmental characteristics with oviparous reptiles – however, show that higher incubation temperatures may cause shorter telomeres in hatchlings (Vedder et al., 2018; Stier et al., 2020; Hope et al., 2022). We showed contrasting results in *L. agilis*, which suggest that either embryo telomere dynamics may be less sensitive to developmental temperature or telomere protection/lengthening mechanisms are more active in this ectotherm than in birds.

While initial telomere length can be indicative of environmental stress experienced by the embryo and thus correlate with offspring condition and early survival (Monaghan and Ozanne, 2018), the rate of telomere change might be a better predictor of prolonged effects (Boonekamp et al., 2014; Sheldon et al., 2022). Moreover, an effect on telomere dynamics is more likely to have long-term implications on overall life history compared with snapshot measurements of telomere length (Watson et al., 2015; Marasco et al., 2022). Our examination of post-hatching telomere dynamics revealed that neonates developed under the warmest treatment were less capable of maintaining the length of their telomeres than those that developed under the two cooler treatments. Higher developmental temperature correlates with growth rate in many reptiles (Noble et al., 2017; While et al., 2018), which is associated with increased oxidative stress and telomere shortening (Alonso-Alvarez et al., 2007; De Block and Stoks, 2008; Smith et al., 2016). This may overwhelm the telomere-restoring processes in neonates, causing telomere erosion. Surprisingly, the effect of developmental temperature on neonate telomere dynamics could not be explained by differential growth as there was no relationship between the absolute change in neonate telomere length and their increase in body size. This suggests that neonates may, indeed, compensate for the telomere shortening commonly associated with developmental growth (reviewed in Monaghan and Ozanne, 2018). A similar response was recently observed in salmonid neonates, in which cell proliferation rate changed with developmental temperature while not influencing telomere shortening (McLennan et al., 2018). Another potential mechanism by which developmental temperature may alter post-hatching telomere dynamics is by affecting the antioxidant capacity of neonates. This was shown in red-eared slider turtle (*Trachemys scripta elegans*) hatchlings, in which higher temperatures caused a reduction in the antioxidant capacity of neonates (Treidel et al., 2016), which has the potential to increase the rate of telomere shortening through increased oxidation (von Zglinicki, 2002). Prolonged effects on antioxidant defence could therefore have important implications on lifetime telomere dynamics. A third mechanism that could explain our results is that the rearing conditions of neonates were the same for all offspring, and closest to the coolest incubation treatment. Thus, cool-incubated offspring may be more suited, through developmental programming (for reviews, see Beaman et al., 2016; Singh et al., 2020), to the conditions in the laboratory. Negative physiological responses (including oxidative stress and telomere shortening) may therefore be greatest in warm-incubated offspring reared in a cooler environment.

We showed that neonate sex was the strongest predictor of telomere dynamics. Specifically, males and females differed both in their telomere length at hatching and in the change in telomere length across the early post-hatching period. Such dimorphisms are a common occurrence (reviewed in Barrett and Richardson, 2011); however, the direction of sex effects varies among, and within, taxa (Remot et al., 2020). Furthermore, a recent meta-analysis revealed that the homogametic sex is generally the longer-lived sex (Xirocostas et al., 2020). This trend was strongest in species with male heterogamety, while the directionality of sex bias was more variable in taxa that include female heterogamety such as birds, reptiles and fish. Life history theory predicts that the sex receiving the greatest fitness returns from late-life reproduction should invest more in maintenance early in life. Species with female heterogamety, including *L. agilis*, therefore offer important opportunities to study the evolution of sex-specific telomere dynamics and life history traits. In this study, females hatched with longer telomeres than those of males and on average lengthened their telomeres post-hatching, whereas males' telomeres shortened. These results are in contrast to our recent findings in another population of *L. agilis* suffering from low genetic variation, in which male hatchlings had longer telomeres than females (Olsson et al., 2022). Although the variation in telomere length was only about 4% – compared with 34% favouring females in the current study – it clearly highlights the complexity of predicting early-life telomere dynamics in ectotherms. Nevertheless, the sexual dimorphism in telomere dynamics observed in the present study may be explained by life history theory and sex-specific reproductive strategies. Females live longer than males and have a size-dependent fecundity (Olsson, 1988; Strijbosch and Creemers, 1988; Olsson and Shine, 1996), which in species with indeterminate growth generally translates to an increased fecundity with age. Selection should therefore favour longevity that promotes later-life reproduction with greater relative contribution to lifetime reproductive success. In contrast, selection might favour a ‘faster’ life history in male *L. agilis*, in which selection drives these polygynous males to invest in traits that maximise current partner acquisition and reproductive success while neglecting somatic maintenance that promotes longevity (Clutton-Brock and Isvaran, 2007). Such selection pressures are likely to be strengthened by the energetically costly reproductive behaviours in male *L. agilis*, including mate competition through guarding, aggression, sperm competition and sexual ornamentation (Olsson, 1994; Olsson et al., 1996a,b; Lindsay et al., 2016).

Combined, our results suggest that early-life telomere dynamics may constitute subtle markers of sex-specific reproductive strategies that will manifest as effects on survival and fecundity later in life, driven by differential investments in early-life maintenance. This is supported by previous work on adult *L. agilis*, which showed that females have longer telomeres and invest more in telomere maintenance, and that telomere length was a stronger predictor of lifetime reproductive success and lifespan in females compared with males (Olsson et al., 2011a). A similar sex-specific effect of early-life telomeres on lifetime reproductive success, linked to an increased lifespan, was recently reported in house sparrows, *Passer domesticus* (Heidinger et al., 2021). We, therefore, hypothesise that the observed sexual dimorphism in early-life telomere dynamics of *L. agilis* is a consequence of sex-specific life histories evolved through trade-offs between current and future reproductive success. Specifically, females are expected to invest more, compared with males, in somatic maintenance early in life to maximise lifetime reproductive success by following a longevity-promoting life history trajectory. Future research is needed to determine the role of telomeres in life history trade-offs and whether telomere maintenance is costly and institutes a constraining factor on current–future life history trade-offs.

Maternal telomere length did not affect initial offspring telomere length but importantly offspring telomere maintenance capacity was positively associated with maternal telomere length. This may simply be due to higher-quality females having longer telomeres and producing high-quality offspring capable of greater investment in life history traits across the board (Hamel et al., 2009; Wilson and Nussey, 2010). Few non-human studies have examined the relationship between maternal telomere length and the capacity for telomere maintenance in offspring. However, Cowell et al. (2021) and Martens et al. (2021) showed that children born from mothers with longer telomeres had lower rates of telomere attrition during childhood. Moreover, mutations in genes that regulate the expression of telomerase have been shown to be heritable in humans and mice, and thereby may influence the telomere maintenance capacity of the next generation (Calado and Dumitriu, 2013; Blackburn et al., 2015). Longer telomeres in female *L. agilis* may, therefore, be indicative of increased telomerase activity, and if heritable would explain why their offspring have increased telomere maintenance capacity. This relationship is predicted by selection when telomere length is associated with lifetime reproductive success, and telomere elongation is costly and constrained under a current–future life history trade-off. Long maternal telomeres may signal for a low-mortality environment and favourable resource availability allowing for telomere elongation. Thus, selection should drive resource allocation away from current reproductive gains and towards somatic maintenance that promotes longevity (Eisenberg and Kuzawa, 2018). Finally, we showed that longer initial telomeres shortened at a faster rate compared with short telomeres, even after correcting for regression to the mean (Verhulst et al., 2013). This result can be explained mechanistically and evolutionarily; firstly, longer telomeres have a greater surface area and more guanine bases and thus become more sensitive to oxidation (Henle et al., 1999; Oikawa et al., 2001). Secondly, stabilising selection can act on very short and very long telomeres, the first being associated with a reduction of homeostasis and diminished survival, while the latter increases the risk of immortalising cancer cells (Aviv et al., 2017; Young, 2018). However, the association between telomere length and organismal fitness, and thus selective pressure, varies greatly across taxa (for reviews, see Olsson et al., 2018a,b).

In conclusion, we show that telomere maintenance capacity is negatively related to developmental temperature, and that early-life telomere dynamics are strongly sex specific in *L. agilis*, with females having longer telomeres and elongation and males having shorter telomeres that shorten further. We show a remarkable capacity for telomere maintenance where, regardless of sex, telomere length was not influenced by early rapid growth. The rate of telomere change was inversely related to initial telomere length and, contrary to expectations, with an initial telomere length about 34% longer than that in males, females still maintained their telomeres considerably better. These results suggest that females invest enough resources in telomere maintenance to achieve telomere elongation despite potential high rates of shortening due to rapid cell proliferation and having long telomeres. Such investments are probably explained by strong selective pressures on longevity in *L. agilis* females in which fecundity is positively related to size/age. This system offers exciting opportunities to study the long-term effects of early-life environments on telomere dynamics and their role in the evolution of sex-specific life histories.

## Supplementary Material

10.1242/jexbio.246512_sup1 Supplementary information

Table S1. Dataset 1
