# Supplementary material for: Sex and early-life conditions shape telomere dynamics in an ectotherm
Source: J Exp Biol. 2024 Feb 9;227(3):jeb246512. doi: 10.1242/jeb.246512 (PMC10912812; doi:10.1242/jeb.246512)
Supplement: Supplementary information [file jexbio-227-246512-s1.pdf]

Table S1. Dataset 1

Available for download at
https://journals.biologists.com/jeb/article-lookup/doi/10.1242/jeb.246512#supplementary-data

Journal of Experimental Biology • Supplementary information
